# Multi-omics cluster defines the subtypes of CRC with distinct prognosis and tumor microenvironment

**DOI:** 10.1186/s40001-024-01805-8

**Published:** 2024-03-28

**Authors:** Yuan Ma, Jing Li, Xu Zhao, Chao Ji, Weibin Hu, YanFang Ma, Fengyi Qu, Yuchen Sun, Xiaozhi Zhang

**Affiliations:** Department of Radiation Oncology, The First Affiliated Hospital of Xi’an Jiaotong University, Yanta West Road 277, Xi’an, 710061 Shaanxi China

**Keywords:** Colorectal cancer, Multi-omics profile, Molecular subtype, Prognostic marker, MID2

## Abstract

**Background:**

Colorectal cancer (CRC) is a complex malignancy characterized by diverse molecular profiles, clinical outcomes, and limited precision in prognostic markers. Addressing these challenges, this study utilized multi-omics data to define consensus molecular subtypes in CRC and elucidate their association with clinical outcomes and underlying biological processes.

**Methods:**

Consensus molecular subtypes were obtained by applying ten integrated multi-omics clustering algorithms to analyze TCGA-CRC multi-omics data, including mRNA, lncRNA, miRNA, DNA methylation CpG sites, and somatic mutation data. The association of subtypes with prognoses, enrichment functions, immune status, and genomic alterations were further analyzed. Next, we conducted univariate Cox and Lasso regression analyses to investigate the potential prognostic application of biomarkers associated with multi-omics subtypes derived from weighted gene co-expression network analysis (WGCNA). The function of one of the biomarkers MID2 was validated in CRC cell lines.

**Results:**

Two CRC subtypes linked to distinct clinical outcomes were identified in TCGA-CRC cohort and validated with three external datasets. The CS1 subtype exhibited a poor prognosis and was characterized by higher tumor-related Hallmark pathway activity and lower metabolism pathway activity. In addition, the CS1 was predicted to have less immunotherapy responder and exhibited more genomic alteration compared to CS2. Then a prognostic model comprising five genes was established, with patients in the high-risk group showing substantial concordance with the CS1 subtype, and those in the low-risk group with the CS2 subtype. The gene MID2, included in the prognostic model, was found to be correlated with epithelial–mesenchymal transition (EMT) pathway and distinct DNA methylation patterns. Knockdown of MID2 in CRC cells resulted in reduced colony formation, migration, and invasion capacities.

**Conclusion:**

The integrative multi-omics subtypes proposed potential biomarkers for CRC and provided valuable knowledge for precision oncology.

**Supplementary Information:**

The online version contains supplementary material available at 10.1186/s40001-024-01805-8.

## Introduction

Colorectal cancer (CRC) is a major global health concern, which ranks third in morbidity (10.0%) and second in mortality (9.4%) worldwide, with an estimated 1.9 million new cases and 935,000 deaths yearly [1]. There are large differences in survival rate depending on stage of disease at diagnosis [2]. For patients with localized CRC, the 5-year survival rate is about 90%. However, approximately 20% of patients already at an advanced stage at the time of diagnosis, the 5-year survival rate drops to 12.5% [2]. Besides, the molecular heterogeneity can result in different outcomes for patients even with similar clinicopathological features [3]. To date, increasing evidence has certified that biomolecules hold great promise in predicting disease prognosis and identifying potential treatment targets. Hence, the molecular subtyping of CRC is urgently needed.

Multi-omics data refer to the amalgamation of transcriptomic, genomic, and epigenetic information that can provide a more comprehensive understanding for cancer heterogeneity. Multi-omics-based classification can help identify the most relevant biomarkers and treatment targets for various types of tumors [4–8]. The initiation and progression of CRC are driven by a series of aggressive gene mutations and epigenetic alterations [9]. By studying the multi-omics, which refers to the analysis of various biological molecules, we can gain a more holistic view of the biological characteristics underlying CRC [10]. An integrative multi-omics study revealed that early-onset CRC have higher tumor mutation burden and different biological and clinical features from late-onset CRC [11]. The CRC Subtyping Consortium proposed classic consensus molecular subtypes (CMS), which have distinct gene expression profiles, genomic alterations, immune infiltrations and therapy responses [12].

Cancer is a complex disease with high heterogeneity, the occurrence of CRC undergoing multiple gene mutations and epigenetic modifications such as DNA methylation [13–15]. DNA methylation and somatic mutation can strongly perturb gene expression [11, 16]. A meta-analysis showed that KRAS, BRAF and p53 mutations were associated with the lymphatic and distant metastases of CRC [17]. A systematic review indicated a 1.49-fold greater risk of colorectal cancer in BRCA1 mutation carriers [18]. Changes in DNA methylation also can serve as biomarkers for the diagnosis, prognosis, and treatment response of CRC [19]. Hypomethylation is observed from early adenomas to metastases, with a linear correlation between demethylation grade and disease stage [20]. LINE-1 hypomethylation is a unique feature of early-onset colorectal cancer and inversely correlated with microsatellite instability (MSI) and CpG island methylator phenotype [21, 22]. The hypermethylation of MGMT, a DNA repair enzyme, is associated with chemotherapy response in metastatic CRC [23, 24].

This study will discuss the use of combinatorial algorithms and multi-omics data in defining the different CRC molecular subtypes and their associated prognostic implications. We identified two distinct subtypes with distinct prognosis and validated with Gene Expression Omnibus (GEO) datasets. Specifically, we also comprehensively depicted the functional annotations, immune status, somatic mutations, copy number variations (CNV), and gene expression patterns of distinct subtypes. We also developed a risk model based on the subtypes related genes and subsequently conducted in vitro experiments to validate the function of the identified gene.

## Methods

### Data source and preprocessing

Molecular profiles of CRC patients were retrieved from The Cancer Genome Atlas (TCGA) using the “TCGAbiolinks” R package for the multi-omics data analysis. A total of 510 CRC patients with complete RNA-seq profiles, miRNA-seq profiles, the Illumina 27 K and 450 K DNA methylation, somatic mutations, and clinicopathological features were selected for subsequent analysis. And the RNA-seq were converted to the log2 “transcripts per million (TPM)” format for subsequent analysis.

Gene expression profiles of datasets (GSE39582, GSE17538, and GSE41258) with RNA expression data and survival information were downloaded from the GEO database for external validation using the “GEOquery” R package.

### Identification of subtypes through integrative multi-omics analysis

To perform clustering with “MOVICS” R package [25], the CRC multi-omics data (mRNA, lncRNA, miRNA, DNA methylation CpG sites, and somatic mutation data) were transformed to features in rows and samples in columns. There are 510 CRC patients with complete multi-omics information. Subsequently, we proceeded by selecting the top 50% of variance factors (mRNA, lncRNA, miRNA, DNA methylation CpG sites) with prognostic value (univariate Cox regression analysis, *p* < 0.05), along with genes with mutation frequencies above 0.1, for further in-depth analyses. The clustering prediction index (CPI) and Gaps-statistics based on above multi-omics data were used to determine the optimal number of subtypes. Subsequently, ten clustering algorithms: SNF, PINSPlus, NEMO, COCA, LRAcluster, ConsensusClustering, IntNMF, CIMLR, MoCluster, and iClusterBayes were used to separate CRC patients into different subtypes. Finally, we used “getConsensusMOIC()” function to identify the final clusters with high robustness based on ten clustering methods.

### Evaluation of the activated of signaling pathways

To reveal the function distribution of each subtype, we downloaded 50 tumor-related Hallmark gene sets from Molecular Signatures Database (MSigDB) and 87 metabolism related gene sets from KEGG [26, 27]. The single sample gene set enrichment analysis (ssGSEA) was used to calculated the enrichment scores for each patient, and pathways with different distribution in two subtypes were visualized in heatmaps. “ClusterProfiler” R package was used to evaluate the most significant different enrichment pathways for each subtype based on different expressed genes (DEGs).

### Characterization of genetic alteration on subtypes

We analyzed the mutation landscape through the R package “maftools”. Using the "oncoplot", "OncogenicPathways", "somaticInteractions", "mafCompare", and "plotOncodrive" functions of this package, we analyzed the tumor mutation panorama, base conversion and transversion, amino acid mutation hotspot, mutation frequency of mutation alleles, copy number mutation, and mutual exclusion or coexistence mutation across different subtypes. The somatic copy number alteration (SCNA) data were analyzed using GISTIC2.0 algorithm on GenePattern (https://cloud.genepattern.org/).

### Immune microenvironment analysis and assessment of response to immunotherapy

We also used ssGSEA and xCell to assess the distribution of immunologic functions, immune cells and stromal cells infiltration in each subtype and visualized with boxplots. And boxplots were also utilized to compare the expression of immunological checkpoint. We used the tumor immune dysfunction and exclusion (TIDE) (http://tide.dfci.harvard.edu) web application to estimate the immunotherapy response of each ESCC patient [28]. GSE78220 and IMvigor210 cohorts were used to verify the predictive value of subtype in response to immunotherapy [29, 30].

### External data validation

The “MOVICS” R package was utilized to validate the repeatability of cluster analysis in other CRC cohorts. Firstly, nearest template prediction (NTP) function permits adaptable cross-platform, cross-species, and multiclass predictions, without requiring the optimization of analysis parameters. Then, we compared the prognosis of the predicted cancer subtypes in these validation cohorts.

### WGCNA and prognosis model construction

“WGCNA (Weighted Gene Co-Expression Network Analysis)” R package was used to identify co-expressed gene modules correlated with cluster subtypes. We identified a total of 24 co-expression modules through the topological overlap matrix (TOM) calculation, which were marked with different colors by setting a soft threshold power *β* = 8, which represented genes that shared highly similar expression patterns in CRC patients. Then, the most correlated module was identified after assessing the Pearson correlation coefficient between two subtypes and the co-expression modules. Finally, the genes of module with high trait significance were selected for further analysis.

Genes in target module with significant impact on OS were selected for least absolute shrinkage and selection operator (LASSO) regression analysis. Then, genes strongly correlated with prognosis and their coefficients were obtained. The risk score was calculated by multiplying each obtained coefficient by the corresponding gene expression and summing the total values and patients were stratified into low- and high-risk groups based on the median risk score.

### Cell culture and plasmid transfection

CRC cell lines SW480 and HCT116 were purchased from Shanghai Institute of Cell Biology and cultured with Dulbecco’s modified Eagle’s medium (DMEM; Gibco, USA) containing 10% fetal bovine serum (FBS; Hyclone, USA) at 37℃ 5% CO_2_. To investigate the impact of MID2 on cellular function, the short-hairpin RNA (shRNA) targeting MID2 and control shRNA were purchased from Sangon Biotech (Shanghai, China). Transfections were carried out according to the Lipofectamine 3000 (Invitrogen, USA) protocol.

### Western blot assay

Cells were lysed in RIPA buffer (Beyotime, China), 1 mM PMSF (Beyotime, China), phosphatase inhibitor cocktail (Beyotime, China). The lysates were then denatured in 100℃, separated by 10% SDS-PAGE gel, transferred to PVDF membranes (Millipore, Sigma, USA), and blocked in 5% skimmed milk. The membranes were incubated overnight at 4% with primary antibody: MID2 polyclonal antibody (1:1000, PA5-28457, Thermo Fisher Scientific), GAPDH polyclonal antibody (1:10,000, 10494-1-AP, Proteintech), E-Cadherin polyclonal antibody (1:20,000, 20874-1-AP, Proteintech), N-Cadherin polyclonal antibody (1:2000, 22018-1-AP, Proteintech), Vimentin monoclonal antibody (1:1000, 5741 T, Cell Signaling Technology). Then secondary antibody goat anti-rabbit IgG (1:2000, A0277, Beyotime) was incubated 1 h at room temperature. After washing with TBST solution, the bands were finally visualized using an ECL reagent (Millipore, Sigma, USA).

### Colony formation assay

SW480 and HCT116 cells were inoculated in a six-well plate (800/well). After 10 days, the cells were fixed with 4% paraformaldehyde and stained with 1% crystal violet. The number of visible colonies was counted to evaluate the colony formation ability of the cells.

### Transwell assay

Cell migration and invasion were measured using Boyden chambers in 24-transwell plates (8 μm pores, Corning). 600 μL DMEM medium containing 20% FBS were added to the bottom of plates. Then, 2.5 × 10^4^ cells suspended in a 200 μl serum-free medium were seeded to the upper chambers for migration assay, 5 × 10^4^ cells were seeded in upper chamber pre-coated with 60 μL of Matrigel (BD) for invasion assay. After incubation for 24 h at 37 °C, the membranes were fixed with 4% paraformaldehyde and stained with 1% crystal violet.

### Statistical analysis

GraphPad Prism (version 8.0, USA) and R language (version 4.2.1) were used for statistical analysis. Student’s t-test and Wilcoxon test were used to compare the discrepancy of continuous data between two groups. Chi-square test and Fisher’s exact test were used to compare the distribution of categorical variable. Kaplan–Meier (K-M) method and log-rank test were used to estimate the survival analysis. *P* value < 0.05 indicated statistical significance.

## Results

### Overview of multi-omics profiling of two CRC subtypes

**Fig. 1:**
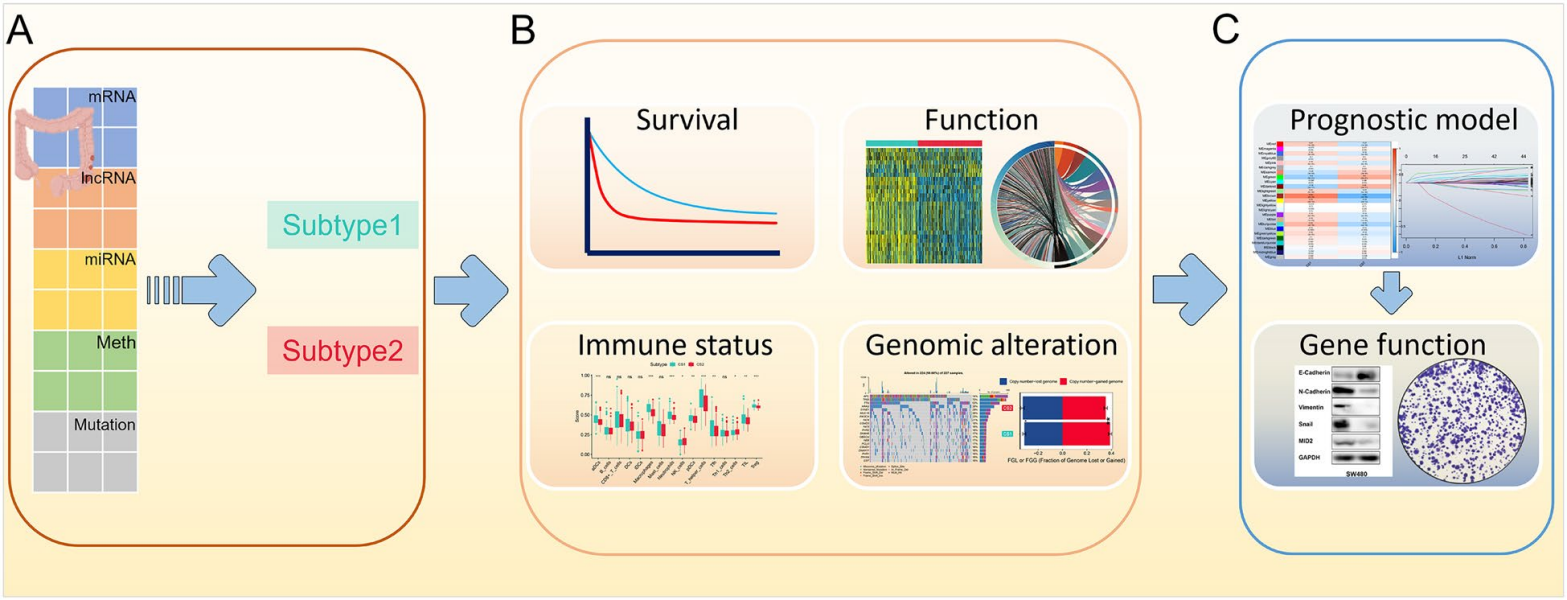
Schematic diagram of the study design. **A** The mRNA, lncRNA, miRNA, DNA methylation CpG sites, and mutation data from TCGA-CRC were systematically organized into comprehensive multi-omics data, which were utilized to identify two subtypes through integrated clustering algorithms. **B** The association of subtypes with prognoses, enrichment functions, immune status, and genomic alterations were further identified. **C** The risk model, constructed through WGCNA and Cox analysis, exhibited substantial concordance with the prognosis of multi-omics subtypes, and the functionality of the molecular marker MID2 in the risk model was validated

**Fig. 2:**
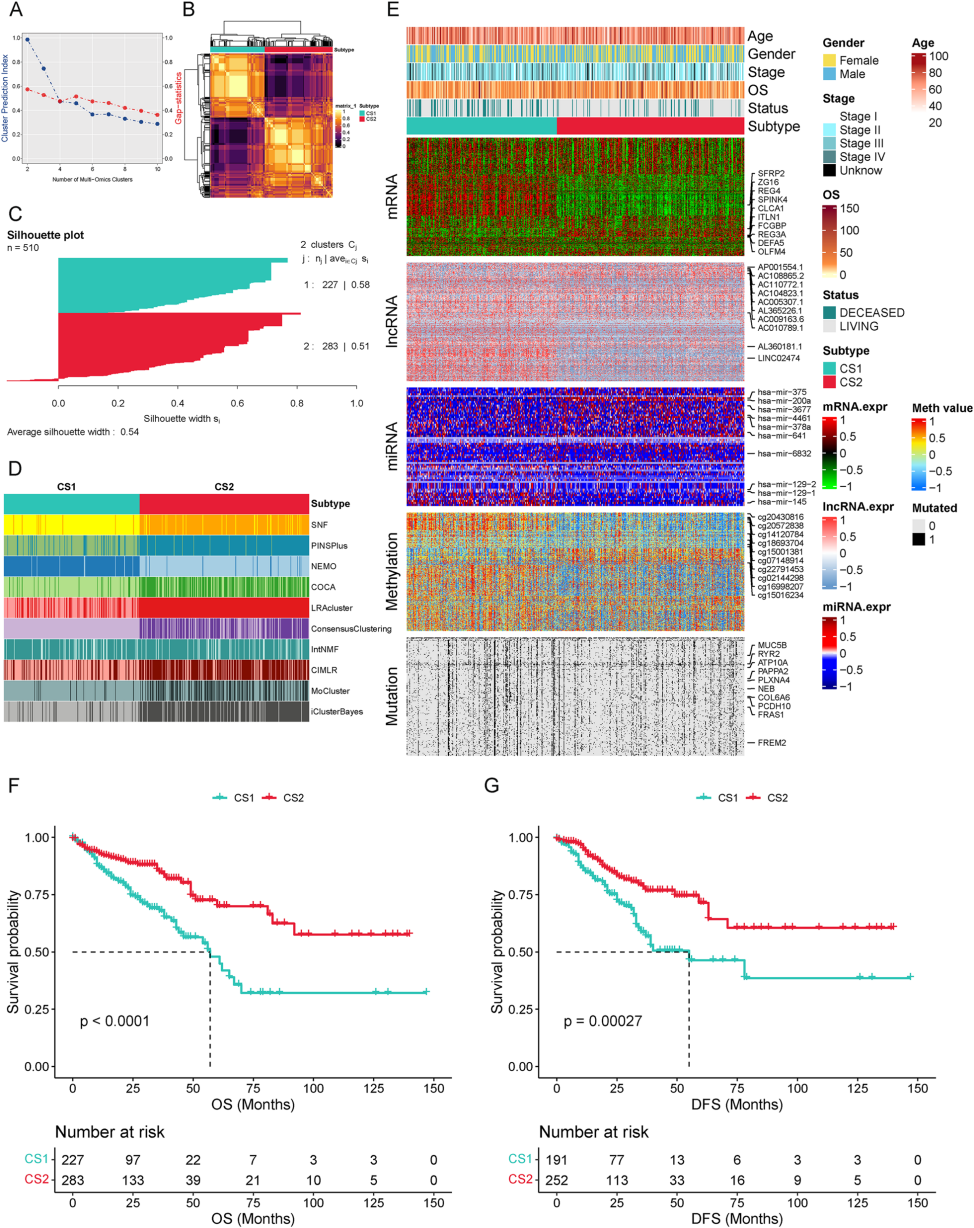
Molecular subtypes clustering based on TCGA-CRC multi-omics data. **A** Prediction of optimal cluster number of multi-omics data by cluster prediction index and Gap-statistics. **B** Consensus heatmap for two cluster subtypes based on multi-omics data. **C** The Silhouette value quantify sample similarity based on two cluster subtypes. **D** Clustering of CRC patients via 10 leading-edge clustering methods. **E** Visualization of multi-omics data for mRNA, lncRNA, miRNA, DNA CpG methylation sites and mutant genes. **F** Differential overall survival outcome in two subtypes. **G** Differential disease-free survival in two subtypes, log-rank test

The flowchart of this study is presented in Fig. 1. We integrated the multi-omics data of TCGA-CRC, with 510 samples having complete multi-omics and survival data used for subsequent cluster analysis. The optimal number of clusters (k = 2) was determined based on the CPI and gap statistics, the number of clusters that reach the maximum sum of these two statistics is considered optimal (Fig. 2A). In comparison to three, four, or five clusters, two clusters showed superior consistency, as confirmed by the consensus matrix (Fig. 2B, Additional file 1: Fig. S1A, B, and C). The Silhouette value, a clustering quality indicator to assess the effectiveness of clustering for each data point ranging from − 1 to 1, demonstrated that the high silhouette width of the two clusters (0.58 and 0.51), which was closer to 1 than the other clusters, represented the robustness of two subtypes and indicated better clustering performance (Fig. 2C, Additional file 1: Fig. S1E, F, and G). For consensus clustering, ten independent clustering algorithms refer the uniformity of the multi-omics cluster and we further combined the clustering results via a consensus ensemble approach with “MOVICS” R package (Fig. 2D). The heatmap revealed distinct transcriptomic, genomic, and epigenomic patterns, as well as clinicopathological features of two subtypes (Fig. 2E).

Moreover, the clinical outcomes of patients in the two subtypes were compared. The K-M plots illustrated that the patients in CS1 had worse prognoses than CS2 (OS: *p* < 0.0001; DFS: *p* = 0.00027; Fig. 2F, G). Table 1 shows the demographic features of CRC patients in the TCGA cohort. Notably, CS2 had more N0, T1–2, and I stage, while CS1 was associated with longer tumor dimension, with an average size of 1.38 ± 0.61 cm, compared to CS2 is 1.17 ± 0.50 cm (*p* < 0.001). And multivariable Cox regression analysis indicates that the impact of multi-omics subtypes on survival is independent of other clinicopathological factors (Additional file 2: Fig. S2).

**Table 1:** The clinicopathological parameters of colorectal cancer patients in TCGA

Subtype	CS1 (n = 227)	CS2 (n = 283)	*p*
Age	64.71 ± 13.44	66.09 ± 12.53	0.236
Gender (%)			
Female	107 (47.1)	136 (48.1)	0.906
Male	120 (52.9)	147 (51.9)	
M stage (%)			
M0	160 (70.5)	208 (73.5)	0.654
M1	37 (16.3)	39 (13.8)	
MX	28 (12.3)	31 (11.0)	
Unknown	2 (0.9)	5 (1.8)	
N stage (%)			
N0	114 (50.2)	175 (61.8)	0.005
N1	56 (24.7)	70 (24.7)	
N2	56 (24.7)	38 (13.4)	
NX	1 (0.4)	0 (0.0)	
T stage (%)			
T1	5 (2.2)	14 (4.9)	0.001
T2	24 (10.6)	66 (23.3)	
T3	168 (74.0)	176 (62.2)	
T4	30 (13.2)	26 (9.2)	
Tis	0 (0.0)	1 (0.4)	
Stage (%)			
Stage I	25 (11.0)	66 (23.3)	0.008
Stage II	82 (36.1)	97 (34.3)	
Stage III	74 (32.6)	73 (25.8)	
Stage IV	38 (16.7)	39 (13.8)	
Unknown	8 (3.5)	8 (2.8)	
Cancer type (%)			
Colon adenocarcinoma	137 (60.4)	186 (65.7)	0.247
Colorectal adenocarcinoma	2 (0.9)	0 (0.0)	
Mucinous adenocarcinoma of the colon and rectum	29 (12.8)	28 (9.9)	
Rectal adenocarcinoma	59 (26.0)	69 (24.4)	
Longest dimension (cm)	1.38 ± 0.61	1.17 ± 0.50	0.001
Disease-free status (%)			
Disease free	133 (69.6)	209 (82.9)	0.001
Recurred/progressed	58 (30.4)	43 (17.1)	

### Gene set variation characters of different subtypes

**Fig. 3:**
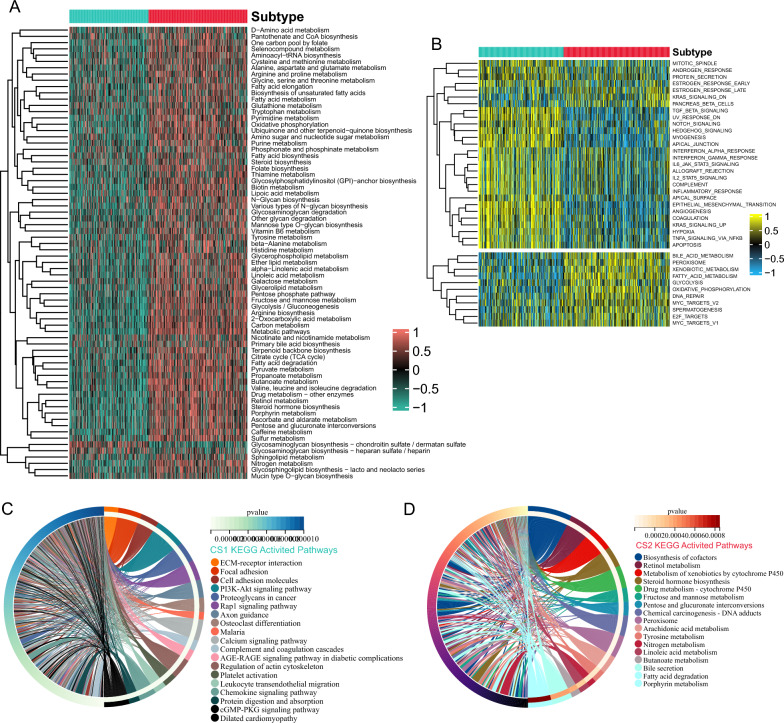
Differential activity of functional enrichment pathways across two subtypes. **A** Heatmap of differentially activated metabolism signaling pathways. **B** Heatmap of differentially activated Hallmark pathways. **C** The circle plot of CS1 subtype activated KEGG pathways. **D** The circle plot of CS2 subtype activated KEGG pathways

Metabolic reprogramming was known to provide valuable insights into metabolic alterations and the mechanisms of disease progression [26, 31]. In this study, we performed metabolic pathway analysis on each patient and observed that CS2 exhibited more metabolic pathway enrichment compared to CS1. Specifically, key metabolic pathways, such as carbon metabolism, TCA cycle, amino acid metabolism, and fatty acid metabolism were upregulated in CS2, while only Glycosaminoglycan biosynthesis-chondroitin sulfate and heparan sulfate were active in CS1, suggesting potentially differences in metabolic profile and energy utilization pattern between the two subtypes (Fig. 3A). To provide a comprehensive overview of the changes in gene expression, we conducted ssGSEA by employing Hallmark gene sets that represent distinct biological states. Figure 3B shows that CS1 was significantly associated with various malignancy pathways, including epithelial–mesenchymal transition (EMT), angiogenesis, hypoxia, TCF-β, and Notch pathways. In terms of KEGG pathways enrichment, we found that CS1 was associated with cell adhesion and other biological characteristics that are indicative of cancer, such as ECM–receptor interaction, focal adhesion, cell adhesion molecules, PI3K–Akt, Rap1 signaling pathway, and so on. And in line with previous findings, CS2 was also enriched in the progression of cellular metabolism (Fig. 3C, D).

### The effect of genetic alteration on subtypes

**Fig. 4:**
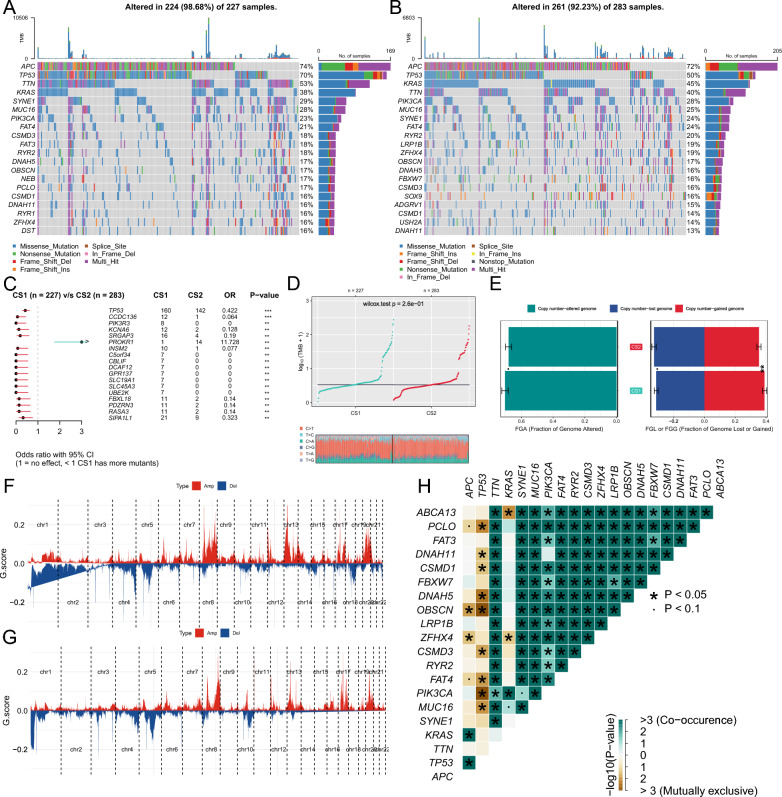
Diversely genetic alterations among two subtypes. **A** The waterfall plot of the top 20 most frequently mutated genes in CS1. **B** The waterfall plot of the top 20 most frequently mutated genes in CS2. **C** Forest plot of significantly differentially mutated genes between two subtypes. **D** Comparison of tumor mutation burden (TMB) and transitions and transversions (TiTv) among two subtypes. **E** Bar plot of fraction genome altered among two subtypes. **F** The copy number amplifications and deletions among the 22 chromosomes in CS1. **G** The copy number amplifications and deletions among the 22 chromosomes in CS2. **H** The heatmap shows the mutually co-occurring and exclusive mutations of the top 20 frequently mutated genes

Gene mutations and copy number alterations are critical events in tumorigenesis and cancer progression. Therefore, we further conducted a deeper analysis on the mutation patterns of different subtypes, and identified genetic alterations that were specifically associated with each subtype. Waterfall plots revealed that several oncogenes and tumor suppressor genes are mutated in overall cohort. The waterfall plot and comparison forest plot showed that CS1 had relatively more genes mutation than CS2. And CS1 had more TP53, TTN, SYNE1, and FAT3 mutation, while CS2 had more KRAS, RYR2, LRP1B, and SOX9 mutation (Fig. 4A, B). Cross-comparisons showed that CS1 had more TP53, CCDC136, PIK3R3, KCNA6 mutation rate, and CS2 only had more PROKR1 mutation rate (Fig. 4C). In oncogene pathway mutation analysis, both two subtype have similar gene in these pathways been affected, while samples in CS1 have higher mutation rates in RTK-RAS, WNT, Hippo, and TP53 pathways (Additional file 3: Fig. S3A, B). We also found that TVP23A, GIPC2, NRAS, RPL22, and KRAS were driver genes in CS1, and TMEM60, FAHD2B, ZNF365, and SHC1 were driver genes in CS2 through the plotOncodrive function of the “maftools” R package (Additional file 3: Fig. S3C, D).

Subsequently, we used the compMut and compFGA function of “MOVICS” R package to investigate the difference of tumor mutation burden (TMB) and genome alteration between two subtypes. There was no significant difference in TMB between the two subtypes (*p* = 0.26), but CS1 had more copy number variations than CS2 (*p* < 0.1, Fig. 4D, E). CS1 had more genomic copy number amplification (*p* < 0.01), and CS2 had more copy number lost (*p* < 0.1, Fig. 4E). We used GISTIC2.0 algorithm to identify recurrent SCNAs present in different subtypes, Fig. 4F and G depict that CS1 had more frequent copy number gains in chromosome regions 1q, 8q, 13q and 20q, and CS2 had more losses in chromosome regions 1p, 5q, 10q, 15p, and 21p. The co-mutation plot revealed most mutation genes are co-occurrence with others, except APC, TP53, and KRAS (Fig. 4H).

### Tumor microenvironment landscape across CRC subtypes

**Fig. 5:**
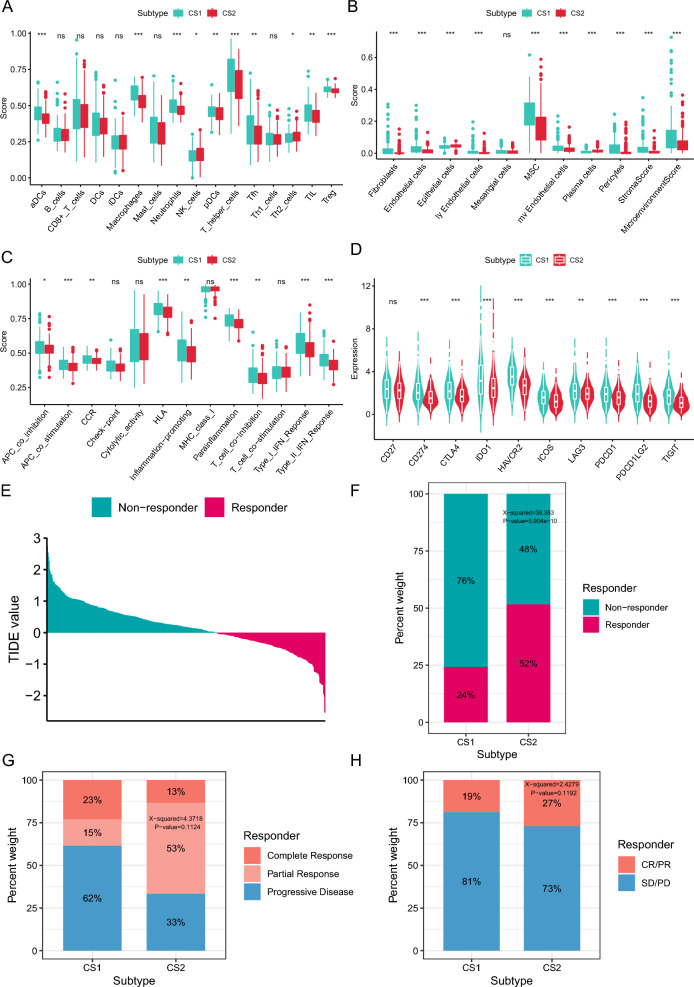
Comparison of immune status across two subtypes. **A** The boxplot of immune cell infiltrations across two subtypes.** B** The boxplot of stroma cell infiltrations across two subtypes. **C** The boxplot of immune functions across two subtypes. **D** The boxplot of the expression of immune checkpoints across two subtypes. **E** The score of immunotherapy response predicted by the TIDE method. **F** The distribution of immunotherapy responders and non-responders across two subtypes. **G** The distribution of immunotherapy response across the nearest template prediction (NTP) predicted subtypes based on GSE78220 cohort. **H** The distribution of immunotherapy response across the NTP predicted subtypes based on IMvigor210 cohort

Tumor microenvironment (TME) is a complex system consisting of various cell types, extracellular matrix, and signaling molecules that play critical roles in tumor development, progression, and metastasis. Therefore, understanding the intricate crosstalk between tumor cells and the TME is essential to develop efficient cancer treatments. Previous results revealed that CS1 was highly enriched in Hallmark pathways related to immune response, such as interferon-α and interferon-γ response, IL-6/JAK/STAT3 signaling, IL2/STAT5 signaling and TNFA via NF-κB signaling (Fig. 3B). There were also significant differences in cellular composition between the two subtypes. CS1 had relatively higher immune cells, such as dendritic cells (DC), macrophages, neutrophils, Th, Tfh, tumor-infiltrating lymphocytes (TILs), and Treg cells, which can drive and regulate T cell-mediated immune responses and interact with each other, while CS2 had more NK cells known as cytotoxic lymphocytes of the innate immune system (Fig. 5A) [32–34]. In terms of stromal cells, CS1 had more fibroblast, endothelial cell, mesenchymal stem cell (MSC), and pericytes that can establish an inflammatory, immunosuppressive and pro-angiogenic microenvironment, while epithelial and plasma cell are more infiltrated in CS2 (Fig. 5B) [35, 36]. In addition, we utilized ssGSEA to examine the differences in immune function and found that most functions, such as APC co-inhibition and co-stimulation, T cell co-inhibition, inflammation promoting, and type I/II IFN response, are active in CS1 (Fig. 5C). Furthermore, we observed higher expression of immune checkpoints (CD274, CTLA4, IDO1, LAG3, PDCD1, and PDCD1LG2) in CS1 compared to CS2 (Fig. 5D).

Then we employed several methods to predict the potential immunotherapy response of patients in different subtypes. A web platform TIDE integrates large-scale omics data to predict immunotherapy response across various tumor types [28]. Therefore, we used TIDE to generate scores reflecting the likelihood of immunotherapy response based on transcriptomic data for each patient (Fig. 5E). The histogram showed that patients in CS2 exhibit a higher probability of responding to immunotherapy compared to those in CS1 (52% vs 24%, *p* = 5.904e−10, Fig. 5F). Subsequently, NTP method was used to predict the multi-omics subtypes of GSE78220 and IMvigor210 cohorts, which provide both immunotherapy response information data and transcriptional data for patients who underwent anti-PD1/PD-L1 therapy [29, 30]. Then we compared the distribution of immunotherapy responses in the two subtypes of patients. While the Chi-square test did not demonstrate statistical significance, Fig. 5G and H illustrate that patient in CS2 exhibited higher rates of immune complete and partial response (GSE78220: 38% vs. 66%, *p* = 0.1124; IMvigor210: 19% vs. 27%, *p* = 0.1192). These results indicate that patients in CS2 may be better suited for immunotherapy.

### Extra validation for molecular subtypes in GEO cohorts

**Fig. 6:**
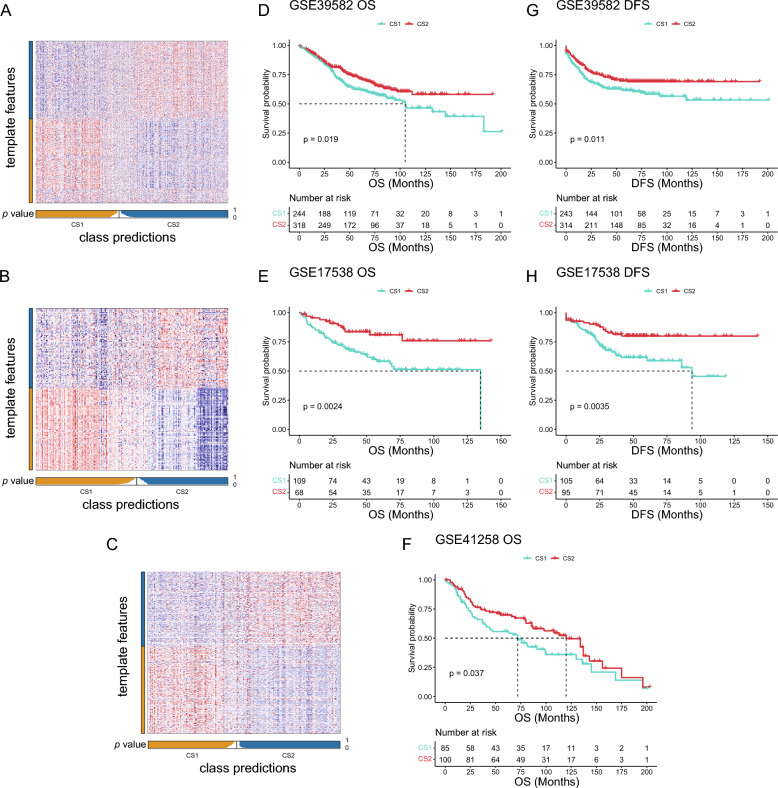
Validation of the multi-omics subtypes in external cohorts. **A–C** Heatmap of NTP in GSE39582 (**A**), GSE17538 (**B**), GSE41258 (**C**) cohorts using subtype-specific upregulated biomarkers identified from the TCGA-CRC cohort. **D-F** Differential overall survival outcome in two subtypes in external cohorts. **G-H** Differential disease-free survival in two subtypes in external cohorts

To validate the molecular subtypes identified through our multi-omics analysis, we included external CRC cohorts with transcriptome and follow-up information for further analysis. Three external cohorts (GSE39582, GSE17538, and GSE41258) were downloaded and prepared for validation. Using the "limma" package, we identified the top 200 upregulated genes in CS1 and CS2 subtypes as their marker genes (Additional file 6: Table S1) and applied the NTP method to determine the subtype of each patient in the validation cohorts (Fig. 6A–C). The prognostic predictions for CS1 and CS2 were consistently observed across all three external GEO cohorts, providing robust validation for the molecular subtypes identified in our study (GSE39582: OS *p* = 0.019, DFS *p* = 0.011; GSE17538: OS *p* = 0.0024, DFS *p* = 0.0035; and GSE41258: OS *p* = 0.037, Fig. 6D–H).

### Construction of a prognostic model for multi-omics molecular subtype

**Fig. 7:**
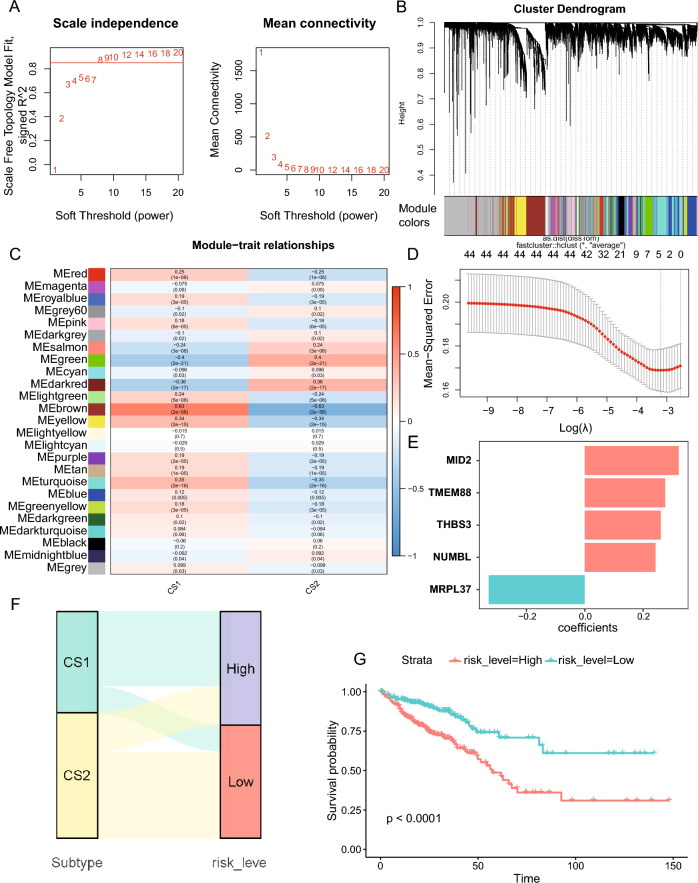
Construction of prognostic model based on genes associated with subtype. **A** Determine the best soft threshold using network topology analysis. **B** The gene dendrogram and module color of weighted gene co-expression network analysis (WGCNA). **C** Heatmap illustrates the correlation between modules and subtypes. **D** LASSO analysis conducted on 44 genes associated with prognosis. **E** The coefficients of the five genes in the prognostic model. **F** The Sankey plot illustrates the alignment between the subtypes and risk groups. **G** Differential overall survival outcome in high- and low-risk groups based on the prognostic model

To enhance the clinical application of the molecular subtype, we aimed to construct a prognostic model for predicting patient outcomes. We utilized the WGCNA to investigate the relationship between gene modules and patient subtypes. Specifically, we constructed gene co-expression networks using the expression profiles of CRC patients, setting *β* at 8 to ensure scale-free networks (Fig. 7A). We then transformed the adjacency matrix into a topology matrix and used the average-linkage hierarchical clustering method to cluster genes, setting the minimum number of genes in each network module to 30. Next, we merged similar gene modules using the dynamic cut tree method, which resulted in 24 distinct modules (Fig. 7B). This analysis allowed us to identify the gene modules most closely associated with the molecular subtype, providing a basis for the development of a robust risk model.

We further calculated the correlations between each module and the subtypes, and found that the brown module had the strongest positive correlation with CS1 and negative correlation with CS2 (Fig. 7C). Next, we performed univariate Cox regression analysis on the 1260 genes in the brown module and identified 44 genes with statistical prognostic significance (*p* < 0.01). To reduce the number of genes and solve multicollinearity problems, we performed LASSO analysis and established a 5-gene prognostic model (Fig. 7D). The final prognostic model is as follows: Risk Score = 0.323 * MID2 + 0.261 * THBS3 + 0.243 * NUMBL + 0.276 * TMEM88 − 0.330 * MRPL37 (Fig. 7E). The CRC patients were then divided into low- and high-risk groups based on the median risk score. And the consistency of the risk model and molecular subtypes was confirmed by the Sankey plot, which showed that CS1 was mainly in the high-risk group and CS2 was in the low-risk group (Fig. 7F). Finally, the K-M survival curve demonstrated a significantly lower survival rate in the high-risk group compared to the low-risk group (*p* < 0.0001, Fig. 7G), indicating a significant difference in prognosis between the two groups.

### MID2 was involved in EMT function in CRC cells

**Fig. 8:**
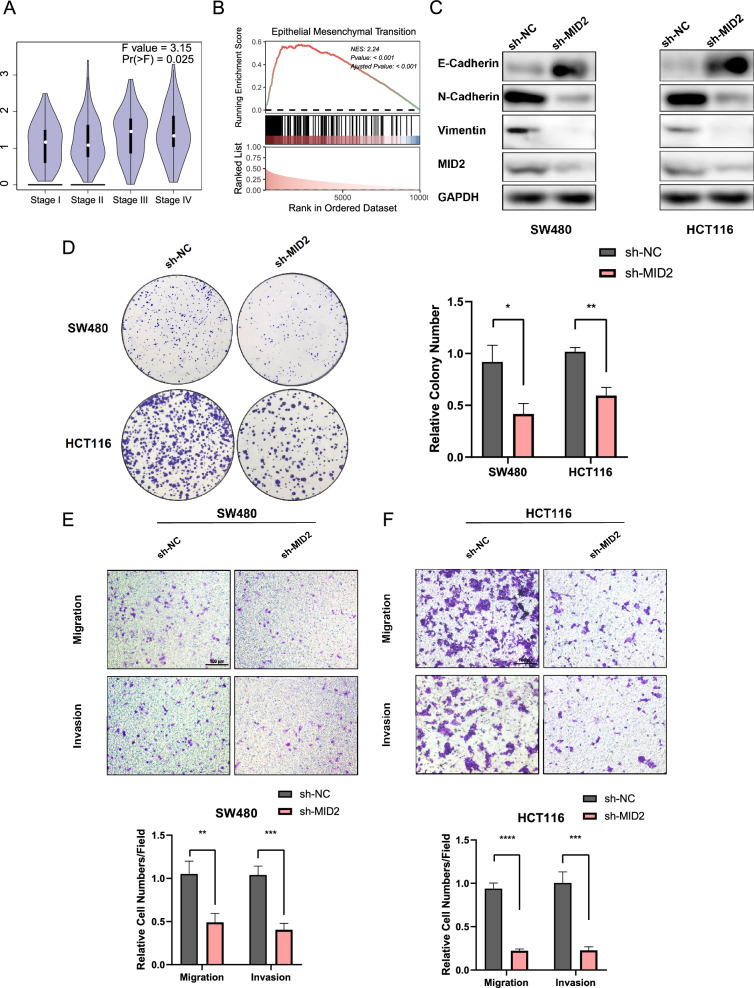
Validation the function of MID2 in CRC cell lines. **A** The expression level of MID2 in different stages of CRC patients. **B** GSEA analysis demonstrates enrichment of MID2-related genes in the epithelial–mesenchymal transition (EMT) pathway. **C** SW480 and HCT116 cells were transfected with plasmid carrying sh-NC or sh-MID2. The protein level of MID2 and EMT biomarkers were assessed using western blotting. **D** Colony formation assay to evaluate the effect of MID2 knockdown on the proliferation of CRC cells. **E–F** Representative images and the comparison of cell migration and invasion ability demonstrated by transwell assay between sh-NC and sh-MID2 cells based on SW480 (**E**) and HCT116 (**F**) cell lines. The scale bar represents100 μm. (*p < 0.05, **p < 0.01, ***p < 0.001, ****p < 0.0001, and two-tailed unpaired t-test)

We observed that MID2, which in previous risk model (Fig. 7E), is a stage-dependent gene, with its expression increasing with CRC stage progression (Fig. 8A). Furthermore, the functional analysis of MID2-related genes using Hallmark gene sets demonstrated a remarkable enrichment in the EMT pathway, with the highest level of significance (Fig. 8B, Additional file 4: Fig. S4). Given our focus on multi-omics research, MID2 displayed a low mutation rate, prompting an investigation into its relationship with DNA methylation. Additional file 5: Fig. S5A indicates that the methylation levels of MID2 CpG sites are not significantly correlated with its expression. Then we explored whether MID2 serves as an upstream regulator of DNA methylation. Additional file 5: Fig. S5B reveals a significant correlation between MID2 and DNA methyltransferase. Additionally, principal component analysis indicates different DNA methylation sites between high and low MID2 expression groups (Additional file 5: Fig. S5C). Previous literatures report the association between DNA methylation in CpG islands and EMT in diseases [37, 38], which implied that the effects of MID2 on cancer progress may be associated with DNA methylation.

Furthermore, we sought to validate the function of MID2 in CRC and confirm the relationship between MID2 and EMT function. Plasmids were used to knockdown the expression of MID2 in SW480 and HCT116 cells. The knockdown efficiency was verified through western blot analysis (Fig. 8C). And the results revealed that the knockdown of MID2 led to upregulation of E-Cadherin and downregulation of N-Cadherin and vimentin (Fig. 8C), which are associated with a mesenchymal phenotype. In addition, we performed the colony formation assay to validate the effect of MID2 on CRC cells proliferation. Figure 8D shows that CRC cells with sh-MID2 had a significantly reduced colony formation capacity compared to cells with sh-NC. Additionally, because EMT is a critical regulator pathway of tumor invasiveness, we assessed the effect of MID2 on cell migration and invasion using transwell assays. As displayed in Fig. 8E and F, knockdown of MID2 significantly inhibited the migration and invasion ability of CRC cells. These findings suggest that MID2 plays a crucial role in the progression of CRC.

## Discussion

CRC is a highly lethal malignancy and ranks as the second leading cause of cancer-related mortality worldwide [1]. Recent advancements in high-throughput biochemical technologies have facilitated the accumulation of multi-omics data, enabling the identification of molecular mechanisms underlying various cancer types. The progression of CRC from colonic epithelium to the development of precancerous polyps, adenomas, and ultimately adenocarcinomas is accompanied by multiple gene mutation, DNA methylation alterations and gene expression changes [39]. Furthermore, it has been observed that early-onset CRC exhibits distinct epigenomic, transcriptomic, proteomic, and metabolic features when compared to late-onset CRC [11, 40]. These findings emphasize the therapeutic and prognostic value of integrating multi-omics data in CRC. In this study, by integrating multiple omics data sets with ten clustering methods, we report two refined and extensively validated CRC subtypes associated with distinct prognosis and molecular characteristics.

In our analysis, CS1 was characterized by poor prognosis and malignant phenotype, such as enriched function of the tumor-related Hallmark pathways, higher genomic alterations, and poor response to immunotherapy. Among the upregulated genes in CS1 (Additional file 6: Table S1), SFRP2 and SFRP4 are Wnt-regulator and can modulate the differentiation of cancer-associated fibroblasts (CAFs), which contribute to the progression and metastasis of the tumor [41–43]. And CAFs can drive FN1, COMP to regulate tumor metastasis and stemness in hepatocellular carcinoma [44, 45]. For CS2 upregulated gene, ZG16 exhibited a sequential decrease from normal to adenoma, and finally to carcinoma in CRC [46]. Furthermore, ZG16 inhibits PD-L1 expression and promote T cell-mediated immunity [47, 48]. And CS2 upregulated gene ITLN1 serve as tumor suppressor factor in various cancers [49]. Besides, we also found ZG16 and ITLN1 were associated with carbohydrate and fat metabolism [50, 51], which is consistent with CS2 enriched in multiple metabolism pathways (Fig. 3A).

It is well known that somatic mutations are present in the genomes of all cancers [52]. Although there was no difference in TMB across the two subtypes, genomic alteration analyses revealed that CS1 had more mutation genes and CNV than CS2. As shown in Fig. 4H, gene mutations are co-occurrence in CRC, and more synchronization mutation genes are present in the CS1 group (Fig. 2E). And one subtype of triple-negative breast cancer has poor prognosis with high chromosomal instability and highly recurrent CNAs [53]. Since various DNA mutagenic-repair events result in gene mutations and CNV [54, 55], tumors with more genomic alteration are suggest phenotypic malignancy and lead to a poor prognosis.

We analyzed immunotherapy responses across different subtypes based on the TIDE web platform and two external independent immunotherapy cohorts. These results implied that patients in CS2 may derive increased benefits from immunotherapy, providing valuable insights to guide clinical drug application. Since it is not convenient to derive the classification from multi-omics data, utilizing transcriptomic data to stratify patients in clinical applications is recommended. For patients identified in CS2 using the NTP approach or classified as low-risk based on the prognostic model in Fig. 7E, immunotherapy is recommended for the higher response rates.

The WGCNA method uses an unbiased systematic approach to analyze biological problems [56], which allows for the construction of gene co-expression networks to help identify candidate biomarkers or therapeutic targets for various diseases [57]. To facilitate the application of the subtypes to clinical practice, we utilized WGCNA to identify gene modular associated with the multi-omics subtypes and constructed a five-gene prognosis model using Cox and LASSO regression analyses. The Sankey plot demonstrated the coincidence of the risk groups with multi-omics subtypes (Fig. 7F). In addition, we found that the expression of MID2 in the prognosis model increase along with tumor stage progression and was significantly associated with EMT function. MID2, which was firstly identified as a causative gene of the X-linked form of a genetic disorder, is a ubiquitin-conjugating E3 enzyme and can regulate cytokinesis [58, 59]. In breast cancer, MID2 is upregulated and mediates tumor chemoresistance [60, 61]. In this study, in vitro experiments proved that MID2 can mediate the proliferation, migration, and invasion abilities of CRC cells, which consistent with the function of MID2 in neural crest cells and previous in silicon results [62]. These results suggest that MID2 is associated with tumor progression and could be a therapeutic target for CRC.

Our research had some limitations. First, the multi-omics subtypes in this study were based on bioinformatic analysis, which needs more time and research to transform into common medical technology. Second, the metabolomics and proteomics data are also critical to understanding cancer, these data might refine the result of multi-omics data analysis. Third, we preliminary validated the biological function of MID2 in CRC, and its detailed molecular mechanisms on EMT function and DNA methylation require future studies.

## Conclusion

In conclusion, our study employed multi-omics data to classify CRC into two subtypes, which were associated with distinct prognoses, enrichment functions, immune microenvironmental characteristics, and altered genomic profiles. Additionally, to facilitate the widespread use of the multi-omics subtypes, a prognostic model based on five genes was constructed that showed strong agreement with the multi-omics subtypes. Besides, through in vitro experiments, we validated the role of MID2, one of the genes in the prognostic model, in promoting EMT function and invasiveness of CRC cells.

### Supplementary Information

**Additional file 1: Figure S1.** (A-C) Consensus heatmap for three (A), four (B), and five (C) subtypes based on multi-omics data. (D-F) The Silhouette value quantify sample similarity based on three (D), four (E), and five (F) cluster subtypes.

**Additional file 2: Figure S2.** Forest plot for multivariable Cox regression analysis with clinicopathological parameters and multi-omics subtypes

**Additional file 3: Figure S3.** (A) Bar plot of oncogenic pathways alterations fraction in CS1. (B) Bar plot of oncogenic pathways alterations fraction in CS2. (C) Scatter plot of the variants fraction of onco-drive genes in CS1. (D) Scatter plot of the variants fraction of onco-drive genes in CS2.

**Additional file 4: Figure S4.** The pathway ranking of MID2-related genes enrichment based on tumor-related Hallmark pathways.

**Additional file 5: Figure S5.** (A) Heatmap of MID2 expression and methylation levels of MID2-associated CpG sites. (B) Heatmap of MID2 and DNA methyltransferases expression. (C) Principal component analysis of DNA methylation patterns in groups characterized by high and low MID2 expression.

**Additional file 6: Table S1.** The top 200 upregulated genes in two subtypes according to nearest template prediction (NTP) method.

## Data Availability

All data generated were shown in this manuscript. TCGA-CRC (https://www.cancer.gov/tcga) and the data sets of GSE39582, GSE17538, and GSE41258 from GEO (https://www.ncbi.nlm.nih.gov/geo/) are publicly open and available.
