# Supplementary figures and images for: Multi-omics cluster defines the subtypes of CRC with distinct prognosis and tumor microenvironment
Source: Eur J Med Res. 2024 Mar 28;29:207. doi: 10.1186/s40001-024-01805-8 (PMC10976740; doi:10.1186/s40001-024-01805-8)

A

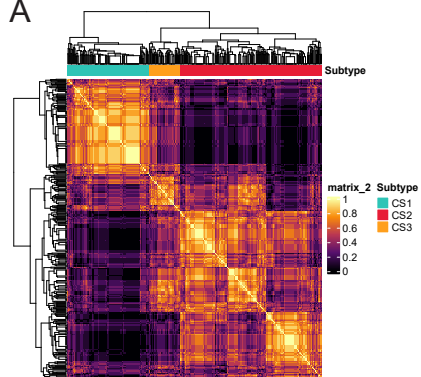

B

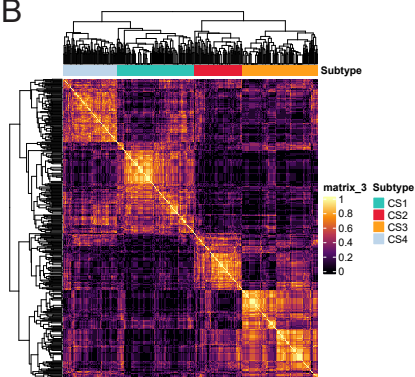

C

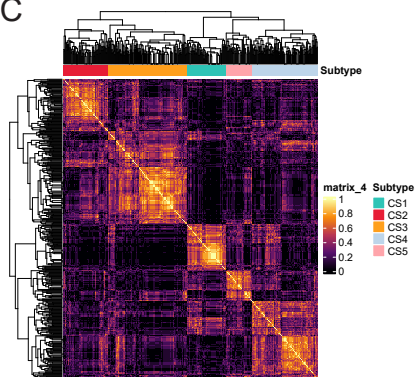

D

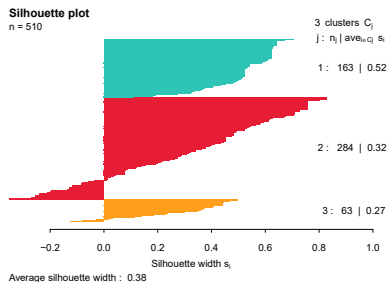

E

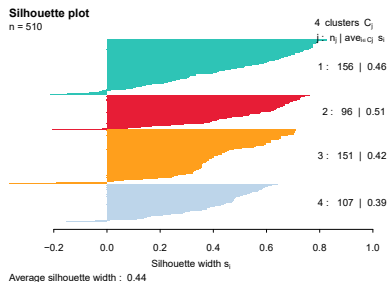

F

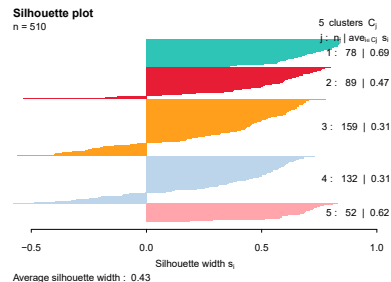

Supplement: Supplementary file 1 — Additional file 1: Figure S1. (A-C) Consensus heatmap for three (A), four (B), and five (C) subtypes based on multi-omics data. (D-F) The Silhouette value quantify sample similarity based on three (D), four (E), and five (F) cluster subtypes. [file 40001_2024_1805_MOESM1_ESM.pdf]

# Hazard ratio

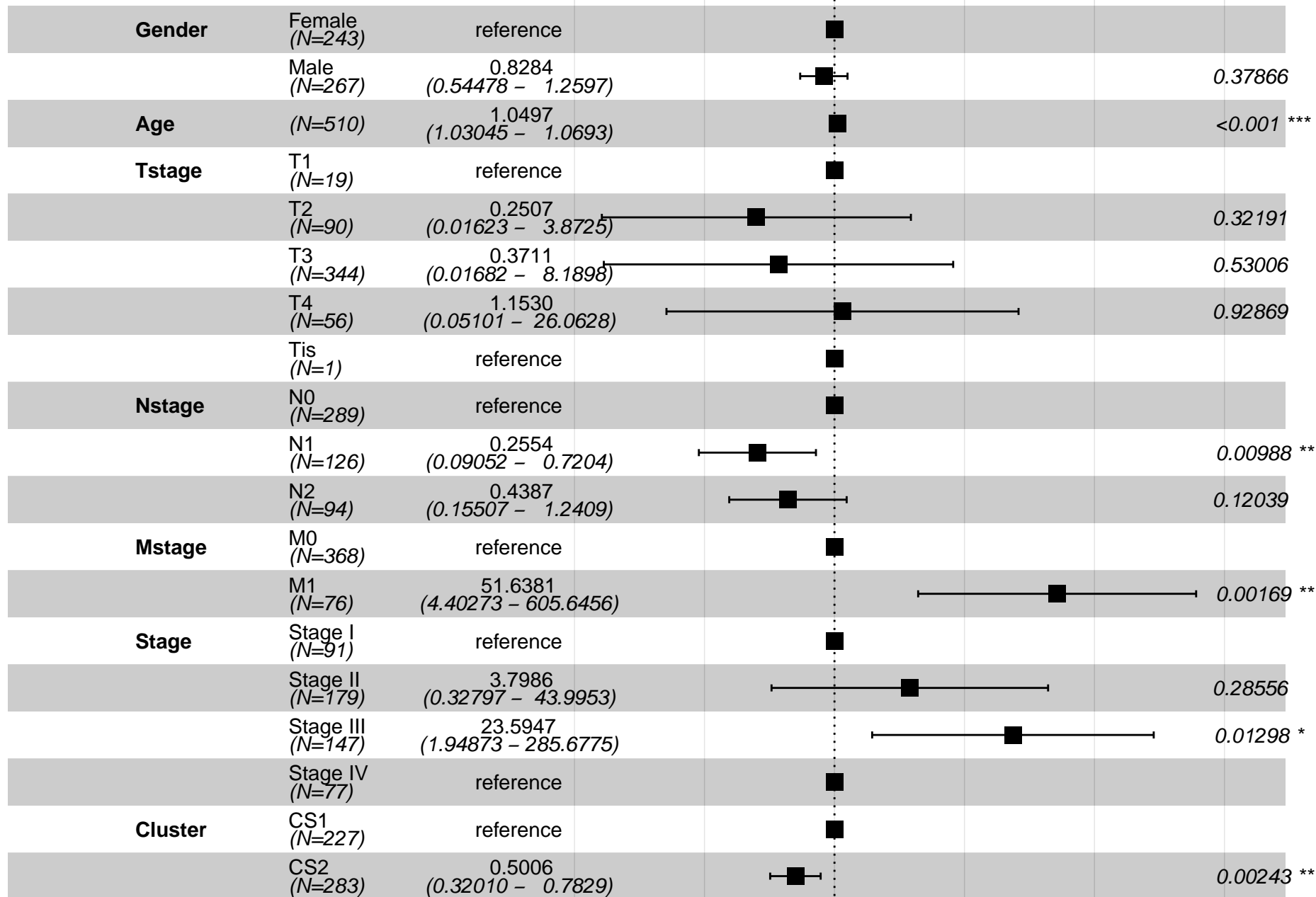

# Events: 94; Global p-value (Log-Rank): 4.5675e-20

AIC: 893.65; Concordance Index: 0.82

0.01

0.1

1

10

100

1000

Supplement: Supplementary file 2 — Additional file 2: Figure S2. Forest plot for multivariable Cox regression analysis with clinicopathological parameters and multi-omics subtypes [file 40001_2024_1805_MOESM2_ESM.pdf]

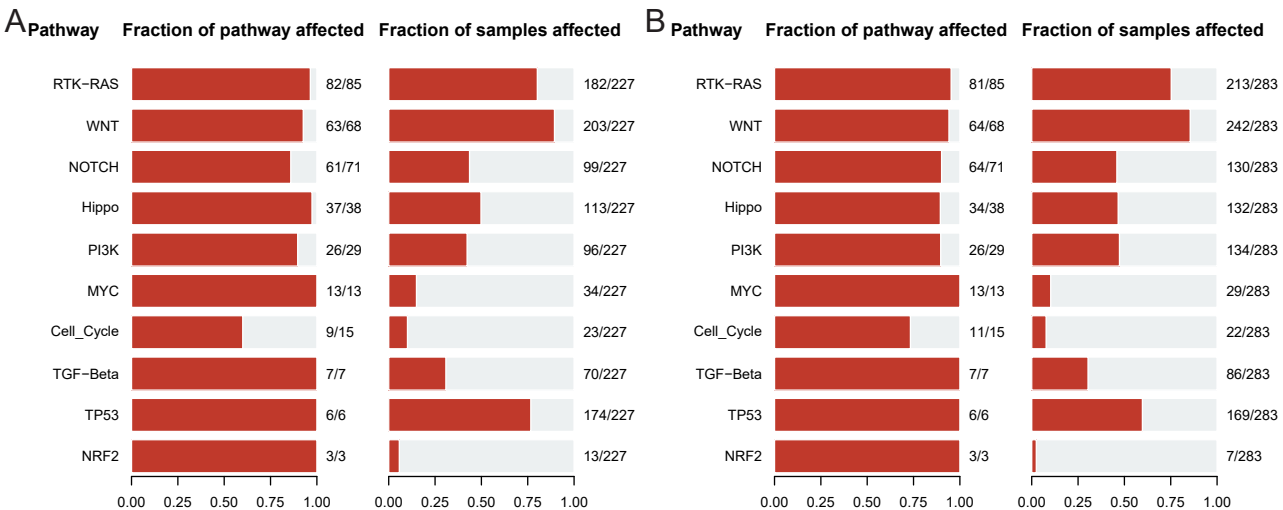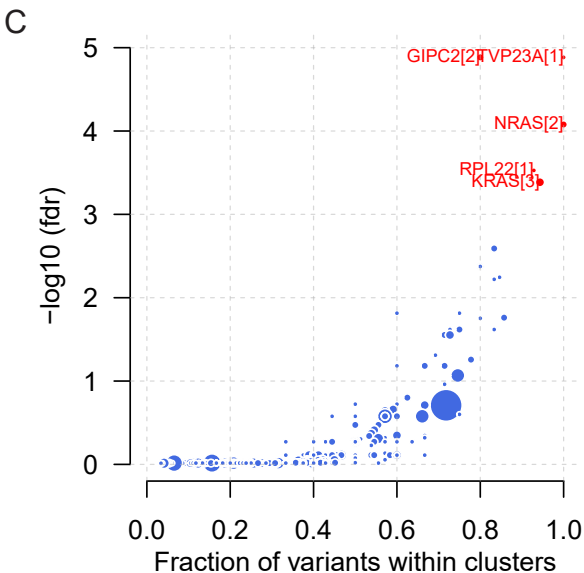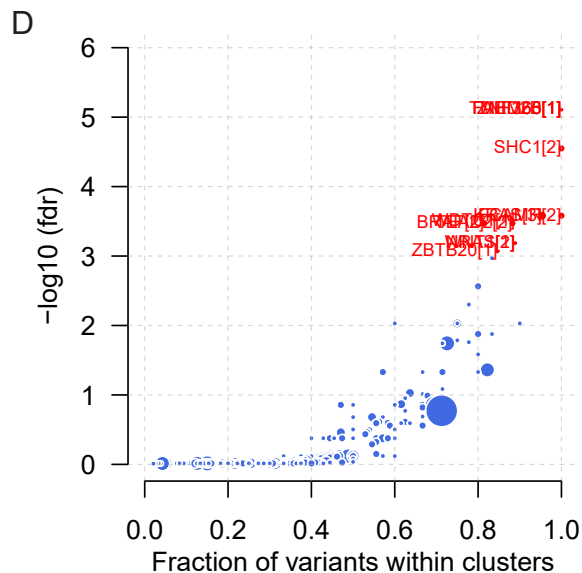

Supplement: Supplementary file 3 — Additional file 3: Figure S3. (A) Bar plot of oncogenic pathways alterations fraction in CS1. (B) Bar plot of oncogenic pathways alterations fraction in CS2. (C) Scatter plot of the variants fraction of onco-drive genes in CS1. (D) Scatter plot of the variants fraction of onco-drive genes in CS2. [file 40001_2024_1805_MOESM3_ESM.pdf]

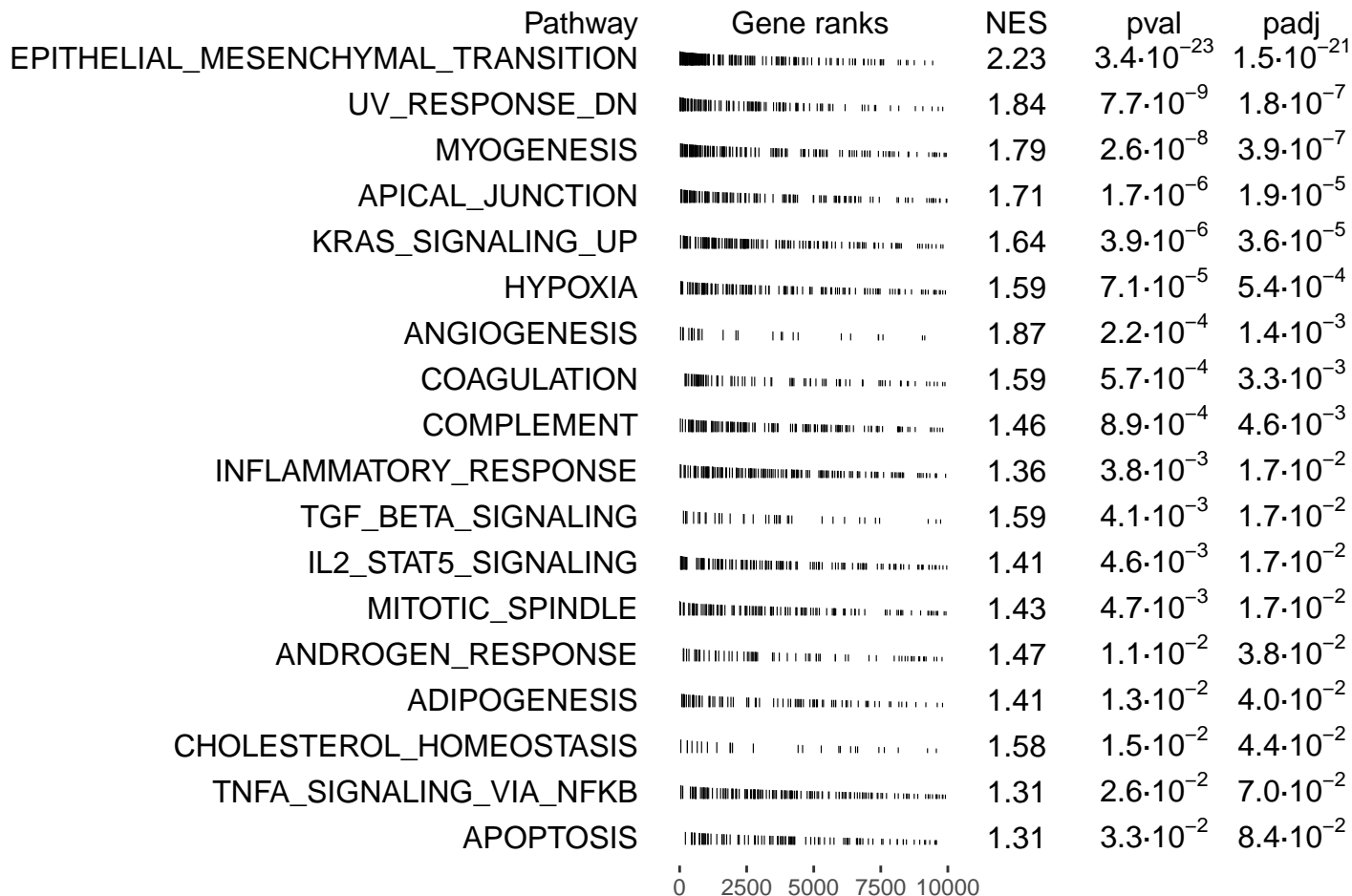

Supplement: Supplementary file 4 — Additional file 4: Figure S4. The pathway ranking of MID2-related genes enrichment based on tumor-related Hallmark pathways. [file 40001_2024_1805_MOESM4_ESM.pdf]

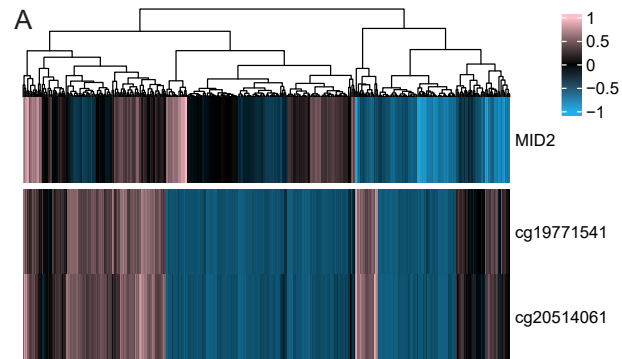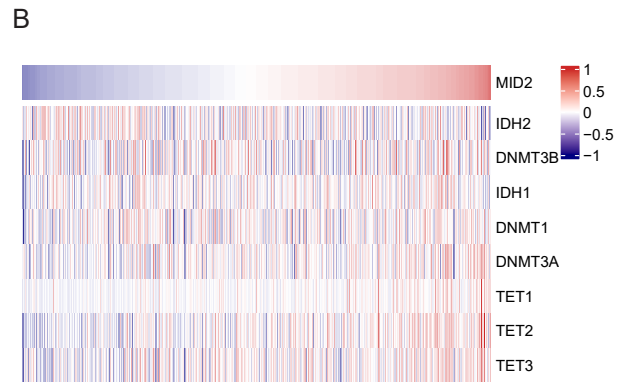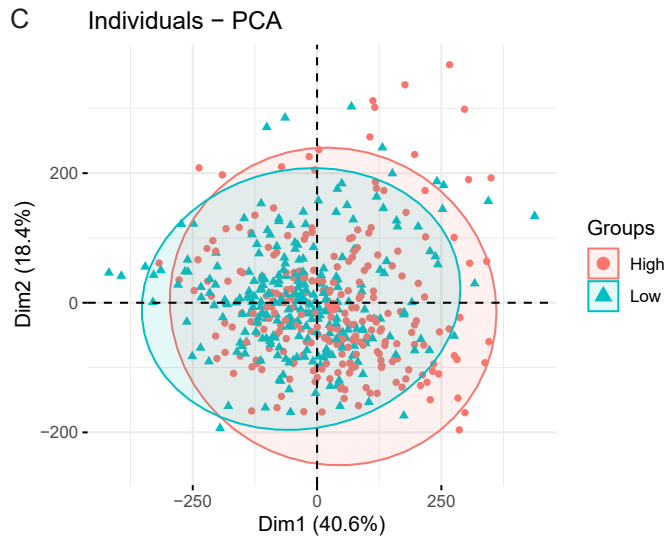

Supplement: Supplementary file 5 — Additional file 5: Figure S5. (A) Heatmap of MID2 expression and methylation levels of MID2-associated CpG sites. (B) Heatmap of MID2 and DNA methyltransferases expression. (C) Principal component analysis of DNA methylation patterns in groups characterized by high and low MID2 expression. [file 40001_2024_1805_MOESM5_ESM.pdf]
