# Supplementary material for: Multi-omics cluster defines the subtypes of CRC with distinct prognosis and tumor microenvironment
Source: Eur J Med Res. 2024 Mar 28;29:207. doi: 10.1186/s40001-024-01805-8 (PMC10976740; doi:10.1186/s40001-024-01805-8)
Supplement: Supplementary file 6 — Additional file 6: Table S1. The top 200 upregulated genes in two subtypes according to nearest template prediction (NTP) method. [file 40001_2024_1805_MOESM6_ESM.pdf]

Table S1 The top 200 upregulated genes in two subtypes according to nearest template prediction (NTP) method

Gene name	Subtype	Direction
SFRP2	CS1	up
FN1	CS1	up
SFRP4	CS1	up
DES	CS1	up
COL10A1	CS1	up
COMP	CS1	up
MYH11	CS1	up
SULF1	CS1	up
POSTN	CS1	up
THBS2	CS1	up
CNN1	CS1	up
SPP1	CS1	up
ASPN	CS1	up
AEBP1	CS1	up
FNDC1	CS1	up
IGFBP5	CS1	up
MGP	CS1	up
GREM1	CS1	up
BGN	CS1	up
COL1A2	CS1	up
COL1A1	CS1	up
CTHRC1	CS1	up
COL3A1	CS1	up
TAGLN	CS1	up
PRELP	CS1	up
CCL21	CS1	up
ANTXR1	CS1	up
LUM	CS1	up
TNS1	CS1	up
TNC	CS1	up
CALD1	CS1	up
COL11A1	CS1	up
COL8A1	CS1	up
SPARCL1	CS1	up
COL6A3	CS1	up
C3	CS1	up
SPOCK1	CS1	up
ACTG2	CS1	up
COL5A2	CS1	up

GPNMB	CS1	up
CCDC80	CS1	up
LMOD1	CS1	up
MYL9	CS1	up
TIMP3	CS1	up
MXRA5	CS1	up
AOC3	CS1	up
RAB31	CS1	up
DPYSL3	CS1	up
AKAP12	CS1	up
TIMP2	CS1	up
PLN	CS1	up
ISLR	CS1	up
SERPING1	CS1	up
MMP2	CS1	up
FBLN2	CS1	up
GAS1	CS1	up
FBN1	CS1	up
VCAN	CS1	up
INHBA	CS1	up
TPM2	CS1	up
C1S	CS1	up
COL5A1	CS1	up
FLNA	CS1	up
ANGPTL2	CS1	up
COL12A1	CS1	up
APOE	CS1	up
EFEMP1	CS1	up
CAVIN1	CS1	up
COL15A1	CS1	up
ADAM12	CS1	up
CHRDL1	CS1	up
SERPINE1	CS1	up
MRC2	CS1	up
DCN	CS1	up
FCGR3A	CS1	up
CCL18	CS1	up
SYNM	CS1	up
SPARC	CS1	up
MARCO	CS1	up
HTRA3	CS1	up
ITGA5	CS1	up
OLFML2B	CS1	up

NEXN	CS1	up
MYLK	CS1	up
CHRDL2	CS1	up
PTGIS	CS1	up
MSRB3	CS1	up
ARL4C	CS1	up
DDR2	CS1	up
HSPB8	CS1	up
CCN2	CS1	up
THBS4	CS1	up
SYNPO2	CS1	up
HSPB7	CS1	up
PDGFRB	CS1	up
COL6A2	CS1	up
FBLN1	CS1	up
C1R	CS1	up
CPXM2	CS1	up
MFAP4	CS1	up
FLNC	CS1	up
EMILIN1	CS1	up
WWTR1	CS1	up
DEPP1	CS1	up
PRRX1	CS1	up
ACTA2	CS1	up
SSC5D	CS1	up
MMP9	CS1	up
CYBRD1	CS1	up
SERPINF1	CS1	up
COL6A1	CS1	up
NNMT	CS1	up
APOD	CS1	up
MMP14	CS1	up
COL14A1	CS1	up
ADAMTS2	CS1	up
FAP	CS1	up
CLU	CS1	up
TGFB3	CS1	up
ITGA11	CS1	up
FSTL1	CS1	up
OLR1	CS1	up
MXRA8	CS1	up
MFAP5	CS1	up
HTRA1	CS1	up

BASP1	CS1	up
TNFAIP6	CS1	up
MMP11	CS1	up
FILIP1L	CS1	up
MEDAG	CS1	up
CCN1	CS1	up
LTBP2	CS1	up
EHD2	CS1	up
NRP2	CS1	up
COL8A2	CS1	up
FIBIN	CS1	up
SORBS1	CS1	up
HEG1	CS1	up
GFPT2	CS1	up
VIM	CS1	up
JCAD	CS1	up
CDH11	CS1	up
NXN	CS1	up
SLC2A3	CS1	up
COL18A1	CS1	up
TREM2	CS1	up
CERCAM	CS1	up
BEX4	CS1	up
FABP4	CS1	up
GGT5	CS1	up
CRABP2	CS1	up
CD163	CS1	up
SDC2	CS1	up
CALB2	CS1	up
ADAMTS12	CS1	up
UCHL1	CS1	up
NTM	CS1	up
MSN	CS1	up
CRISPLD2	CS1	up
RAMP1	CS1	up
LOX	CS1	up
A2M	CS1	up
PLXDC2	CS1	up
PXDN	CS1	up
TMEM47	CS1	up
LRRC15	CS1	up
AQP1	CS1	up
THBS1	CS1	up

COL4A2	CS1	up
GLIS2	CS1	up
HSPG2	CS1	up
NRP1	CS1	up
CRYAB	CS1	up
CHI3L1	CS1	up
CCL2	CS1	up
FERMT2	CS1	up
CTSK	CS1	up
ELN	CS1	up
GEM	CS1	up
MAFB	CS1	up
CLMP	CS1	up
CILP	CS1	up
LHFPL6	CS1	up
GUCY1A1	CS1	up
COL16A1	CS1	up
MYH10	CS1	up
CFH	CS1	up
COL4A1	CS1	up
SMARCA1	CS1	up
ANXA6	CS1	up
CD93	CS1	up
LTBP1	CS1	up
AHNAK2	CS1	up
ATP2B4	CS1	up
APOC1	CS1	up
FHL1	CS1	up
ZEB1	CS1	up
NOTCH3	CS1	up
PPP1R18	CS1	up
SCRN1	CS1	up
PIGR	CS2	up
SPINK4	CS2	up
CLCA1	CS2	up
REG4	CS2	up
OLFM4	CS2	up
ITLN1	CS2	up
FCGBP	CS2	up
MUC2	CS2	up
DUOXA2	CS2	up
PHGR1	CS2	up
ZG16	CS2	up

DEFA5	CS2	up
LCN2	CS2	up
REG3A	CS2	up
CEACAM7	CS2	up
DUOX2	CS2	up
LRRC26	CS2	up
VSIG2	CS2	up
DEFA6	CS2	up
UGT2B17	CS2	up
TFF1	CS2	up
RAB5IF	CS2	up
GUCA2A	CS2	up
REG1A	CS2	up
DMBT1	CS2	up
C10orf99	CS2	up
CA4	CS2	up
FAM3D	CS2	up
JCHAIN	CS2	up
LYPD8	CS2	up
RETNLB	CS2	up
HMGCS2	CS2	up
ATOH1	CS2	up
CKB	CS2	up
NOS2	CS2	up
HEPACAM2	CS2	up
SPDEF	CS2	up
CLCA4	CS2	up
B3GNT6	CS2	up
RNF186	CS2	up
GPA33	CS2	up
ST6GALNAC1	CS2	up
ID1	CS2	up
AKR1B10	CS2	up
AGR3	CS2	up
CCL28	CS2	up
TFF3	CS2	up
PI3	CS2	up
CTSE	CS2	up
TSPAN1	CS2	up
SELENBP1	CS2	up
C4orf48	CS2	up
CA2	CS2	up
PLA2G2A	CS2	up

TPSG1	CS2	up
LCN15	CS2	up
SMIM22	CS2	up
PCBP1	CS2	up
MS4A12	CS2	up
NXPE4	CS2	up
CES3	CS2	up
SPINK1	CS2	up
ENDOG	CS2	up
GNG5	CS2	up
TMEM160	CS2	up
MUC1	CS2	up
ALDOA	CS2	up
TMEM238	CS2	up
PLAC8	CS2	up
AMN	CS2	up
ADH1C	CS2	up
PDZK1IP1	CS2	up
DEGS2	CS2	up
COMTD1	CS2	up
MRPL12	CS2	up
ITM2C	CS2	up
CDC42EP5	CS2	up
TMEM54	CS2	up
FTH1	CS2	up
C15orf48	CS2	up
STYXL2	CS2	up
FABP1	CS2	up
NUDT8	CS2	up
C11orf86	CS2	up
GGT6	CS2	up
MOGAT2	CS2	up
L1TD1	CS2	up
CDX1	CS2	up
ADTRP	CS2	up
GPT	CS2	up
TMEM141	CS2	up
POLR2I	CS2	up
ATP5ME	CS2	up
UQCR11	CS2	up
S100P	CS2	up
WFDC2	CS2	up
ETHE1	CS2	up

LGALS4	CS2	up
RPS10	CS2	up
MEP1A	CS2	up
LEFTY1	CS2	up
HSD11B2	CS2	up
BLOC1S1	CS2	up
NME2	CS2	up
PRELID1	CS2	up
FFAR4	CS2	up
CASP5	CS2	up
KRT20	CS2	up
PXMP2	CS2	up
ELAPOR1	CS2	up
AKR7A3	CS2	up
SMIM32	CS2	up
SERPINA1	CS2	up
NRARP	CS2	up
MUC4	CS2	up
NDUFS7	CS2	up
MRAP2	CS2	up
APOBEC1	CS2	up
C4BPA	CS2	up
GUCA2B	CS2	up
CLDN3	CS2	up
SLC26A3	CS2	up
HHLA2	CS2	up
DHRS9	CS2	up
NAT2	CS2	up
CXCL14	CS2	up
TCN1	CS2	up
CHCHD10	CS2	up
MT1G	CS2	up
SLC25A10	CS2	up
COL9A2	CS2	up
COX8A	CS2	up
C2CD4B	CS2	up
ZC3H12A	CS2	up
MCRIP2	CS2	up
DPEP1	CS2	up
IGFBP2	CS2	up
CAPN9	CS2	up
ERN2	CS2	up
MRPL41	CS2	up

NDUFA11	CS2	up
CXCL3	CS2	up
KLK12	CS2	up
KLF4	CS2	up
UQCR10	CS2	up
NEURL1	CS2	up
BTNL3	CS2	up
UQCRQ	CS2	up
MRPL2	CS2	up
PITX1	CS2	up
LGALS2	CS2	up
NDUFB8	CS2	up
ATP5F1D	CS2	up
LRG1	CS2	up
GCNT3	CS2	up
ACADS	CS2	up
MFSD2A	CS2	up
CLDN2	CS2	up
HYAL1	CS2	up
ENTPD8	CS2	up
DPM3	CS2	up
SULT1B1	CS2	up
ADAT3	CS2	up
LRATD1	CS2	up
IHH	CS2	up
SLC44A4	CS2	up
CASP1	CS2	up
PPP1R14D	CS2	up
NPDC1	CS2	up
GDF15	CS2	up
RABAC1	CS2	up
FABP2	CS2	up
CHP2	CS2	up
PRAC1	CS2	up
ATP5MF	CS2	up
EIF4EBP3	CS2	up
ALKBH7	CS2	up
CDHR5	CS2	up
XDH	CS2	up
LRRC19	CS2	up
PLA2G10	CS2	up
ADH6	CS2	up
CCL20	CS2	up

ANTKMT	CS2	up
COX5B	CS2	up
FOXA3	CS2	up
COX6A1	CS2	up
SNORC	CS2	up
CDHR1	CS2	up
MAZ	CS2	up
REG1B	CS2	up
STARD10	CS2	up
TST	CS2	up
BTNL8	CS2	up
RAB26	CS2	up
B3GNT7	CS2	up
MYO1A	CS2	up
C12orf57	CS2	up
AGR2	CS2	up
TMEM256	CS2	up

3
